# Effectiveness and safety of bleaching agents on lithium disilicate glass ceramics

**DOI:** 10.34172/joddd.2022.040

**Published:** 2022-12-30

**Authors:** Alper Ozdogan, Nihan Kaya

**Affiliations:** ^1^Department of Prosthodontics, Faculty of Dentistry, Atatürk University, Erzurum, Turkey; ^2^Department of Prosthodontics, Faculty of Dentistry, Giresun University, Giresun, Turkey

**Keywords:** Tooth bleaching, Glass ceramics, Dental polishing, Surface properties

## Abstract

**Background.:**

Aesthetic expectations have increased the use of aesthetic materials in dentistry. Lithium disilicates are frequently used materials for these expectations. Bleaching is another method used to provide aesthetics. Bleaching processes on restorative materials are not fully known. This study investigated the effect of at-home and in-office bleaching methods on the color change, surface roughness, and topography of lithium disilicate glass-ceramic materials produced with two different techniques and subjected to different polishing procedures.

**Methods.:**

A total of 144 disc-shaped pressed and computer-aided design (CAD) lithium disilicate glass-ceramic specimens were randomly divided into four groups. Glazing and three different chair-side polishing procedures were performed. The specimens in each group were randomly divided into two groups and subjected to at-home and in-office bleaching processes (n=9). The home bleaching process was repeated with 16% carbamide peroxide agent for six hours for seven days, while the in-office bleaching process was applied with 40% hydrogen peroxide agent for two sessions of 20 minutes. After the bleaching processes, the final color and surface roughness experiments of the specimens were carried out, and the results were recorded. ANOVA and Tukey multiple comparison tests were used FOR the statistical analysis of the data (α=0.05).

**Results.:**

The material*polish*bleaching, polish*bleaching, material*bleaching, and material*polishing interactions were not statistically significant regarding color and roughness changes of both specimens (*P*>0.05).

**Conclusion.:**

Both bleaching processes can be safely applied to lithium disilicate glass-ceramic materials.

## Introduction

The increase in aesthetic expectations has affected dentistry and numerous other fields. The rehabilitation of functional, phonation, and aesthetic discrepancies with prosthetic restorations positively affects the patient’s quality of life. An ideal restorative material should be biocompatible with tooth and bone structures, be in natural harmony with teeth and gingiva, have light transmittance similar to enamel and dentin, and maintain the function of lost or damaged tissues.^1^ Lithium disilicate glass ceramics (LDGCs), which can be produced by both pressing and computer-aided design‒computer-aided manufacturing (CAD-CAM) technology, are frequently preferred in the manufacture of aesthetic restorations today because of their superior aesthetic properties, controllable translucency, flexural strength, and fracture toughness.^2^

In clinical practice, occlusal adjustment is required before the cementation procedure. Although this adjustment is made for aesthetic purposes and to correct inadequate contours, during this process, the glazed surface of the ceramic restoration is removed by abrasion.^3,4^ Although the glazed porcelain surface has been thought to be ideal,^4^ small modifications made on the porcelain surface, especially in cases with time problems, can be corrected with the chair-side polishing method instead of the “reglazing” process.^4^ When the operator cannot check occlusion before bonding due to the low thickness/strength of the material, post-bonding, intraoral occlusal adjustment, and polishing become necessary. Intraoral polishing aims to reduce biofilm retention, discoloration, and discomfort and minimize residual defects that can lead to crack propagation and subsequent biomechanical failure.^5^ Rubber and silicone discs, felts, abrasive stones, and diamond pastes are some materials used for chair-side polishing. All these procedures aim to provide the long-term success of restorations, clinical infection control, and better oral hygiene. In addition, these procedures have the benefit of being effective and fast methods.^6^

Bleaching is a widespread aesthetic treatment option for patients who want whiter, more natural-looking teeth. Although there are other methods to improve dental aesthetics, bleaching is the most preferred option by patients.^7^ Vital tooth bleaching is one treatment performed to remove internal and external discolorations.^8^ At-home and in-office bleaching methods are treatment options to prevent discoloration.^9^ The bleaching agents, such as hydrogen peroxide and carbamide peroxide, are used at different concentrations.^10^ When the bleaching process is applied, not only do the tooth surfaces but also the surface of the pre-existing restorative materials come into contact with the bleaching agents. Although bleaching is safe and effective for dental hard tissues, it may not be safe for restorative materials with corrosive properties and may have different effects on these materials.^11,12^ The incorrect application of the bleaching agent may cause problems on the surface of prosthetic restorations, especially when the bleaching agent is not applied by the patient under the supervision of a dentist, which may affect the clinical duration of the use of restorations.^13^

The color stability of a restoration is as important as the mechanical properties throughout the long-term use of the material. Color changes may limit the longevity and quality of restorations over time.^14^ Changes in the material’s color can be calculated using various parameters and formulas. The color parameters of the materials (L*, a*, b*) have been described by the Commission Internationale de l’Eclairage (CIE) and can be calculated from a transmittance spectrum or reflection using a standard observer, illuminator, and suggested geometry.^15^ The CIEDE2000 color difference formula is superior to the CIELab formula, having the feature of correcting the nonuniformity of the color space in the CIELab, especially for small color differences.^15^ Also, Ghinea et al^16^ showed that the CIEDE2000 color difference formula provides a better fit than the CIELab formula for assessing the acceptability, perceptibility, and color difference thresholds for dental ceramics.

 The current study evaluated the effect of at-home and in-office bleaching methods on the color changes, surface roughness, and topography of LDGC materials produced with two different production techniques and subjected to different polishing procedures. The null hypothesis of the present study was that none of the bleaching and polishing procedures would change the color and surface characteristics of the LDGC materials.

## Methods

 A power analysis was carried out using the G*Power software (v3.0.10) to obtain the highest power level with the smallest specimen size. The analysis showed that at least nine specimens were required for the highest power level (power = 80, α = 0.05), and 144 specimens were used.

###  Specimen preparation

In the present study, 144 LDGC specimens were used (12 × 4 × 2 mm). Seventy-two specimens were produced by pressing from ingots (IPS e.max Press; Ivoclar Vivadent AG, Schaan, Liechtenstein), and the remaining 72 were produced by milling from blocks (IPS e.max CAD; Ivoclar Vivadent AG, Schaan, Liechtenstein). Under the manufacturer’s instructions, the firing processes of the IPS e.max Press (IP) specimens were applied, as indicated in Table 1; the crystallization process of the IPS e.max CAD (IC) specimens was completed at 840°C, and the glaze processes were applied as indicated in Table 1. The specimens belonging to the IP and IC groups were randomly divided into four groups according to their polishing processes and polished with 600-, 800-, and 1200-grit silicon carbide paper under water for 60 seconds to standardize the surface quality. The polishing kits specified in Table 2 were used unidirectionally for 60 s each, with a maximum tip rotating speed of 8000 rpm, according to the manufacturer’s instructions, with an average pressure of 2 N. All procedures were performed by the same operator to apply uniform pressure and application procedures during polishing.

**Table 1:** Description of firing treatments applied to ceramic materials

**Firing program**	**Ingot pressing**	**Dentin firing**	**Glaze firing**
Initial temperature (°C)	700	403	440
Temperature increase rate (°C/min)	60	60	60
Final temperature (°C)	920	750	740
Dwell time at final temperature (min)	25	6.5	6.5

**Table 2:** Instructions and manufacturer information of polishing systems

**Group**	**Material**	**Manufacturer**	**Instruction**
Glaze (G)	IPS e.max Press Ceram Glaze Paste	Ivoclar Vivadent AG, Schaan, Liechtenstein	-
Meisinger (M)	Luster Intraoral Twist Kit	Hager & Meisinger, Neuss, Germany	Respectively; green-blue-red-yellow rubbers without water cooling
OptraFine (O)	OptraFine Assortment;	Ivoclar Vivadent AG, Schaan, Liechtenstein	Respectively; light blue-dark blue under water cooling after OptraFine HP Polishing Paste (Ivoclar Vivadent AG) without water cooling
D + Z (D)	D + Z Polishing Kit	Drendel + Zweilling Diamant GmbH, Kalletal, Germany	Respectively; blue-pink-gray with water cooling

###  Bleaching procedures

After the polishing processes, the specimens were numbered, and the color evaluations of each specimen before bleaching were measured on a gray background in the “single tooth” format of the spectrophotometer (VITA Easyshade V; VITA Zahnfabrik, Bad Sackingen, Germany).^17^ The spectrophotometer probe was settled at the center of the specimens, and measurements were repeated three times. Finally, the average of these three measurements was recorded for each specimen. Then, the specimens were randomly assigned to two groups for the bleaching procedures, and the bleaching procedures were performed according to the manufacturer’s instructions as follows (n = 9):

- *At-home bleaching:* A home bleaching agent (Opalescence; Ultradent Products Inc., South Jordan, Utah, USA) containing 16% carbamide peroxide was applied to the polished surfaces of the specimens using an applicator, by the same clinician, at room temperature (according to the manufacturer’s instructions) and then stored at 37°C during the bleaching period for six hours. Then, the specimen surfaces were washed for one minute and stored in distilled water at 37°C. This process was repeated for seven days.
- *In-office bleaching:* An in-office bleaching agent (Opalescence Boost; Ultradent Products Inc., South Jordan, Utah, USA) containing 40% hydrogen peroxide was applied to the polished surfaces of the specimens for a total of 40 minutes in two 20-minute sessions. Then, the specimen surfaces were washed for one minute and stored in distilled water at 37°C.

After polishing and bleaching procedures, environmental scanning electron microscope (ESEM) imaging of a randomly selected specimen from each group was carried out using an ion beam-SEM tomography electron microscope (Quanta 250 FEG; FEI, Hillsboro, OR, USA) device at × 1000 magnification.

###  Color and roughness evaluation

After bleaching, the color measurements of the specimens were made and recorded with a spectrophotometer (VITA Easyshade V, VITA Zahnfabrik) by applying the color measurement protocols before bleaching. The color differences of the specimens before and after bleaching were calculated using the following formula CIEDE2000 (∆E_00_):

∆E_00 _= [(∆Lʹ/K_L_S_L_)^2^ + (∆Cʹ/K_C_S_C_)^2^ + (∆Hʹ/K_H_S_H_)^2^ + R_T_(∆Cʹ/K_C_S_C_)(∆Hʹ/K_H_S_H_)]^1/2^

where ∆L_0_, ∆C_0_, and ∆H_0_ are the differences in lightness, chroma, and hue between the two specimens that were compared. S_L_, S_C_, and S_H_ are the weighting functions for the lightness, chroma, and hue components, respectively. K_L_, K_C_, and K_H_ are the parametric factors to be adjusted according to different viewing parameters. In the current study, K_L_, K_C_, and K_H_ were set to 1.^18^

**Figure 1:**
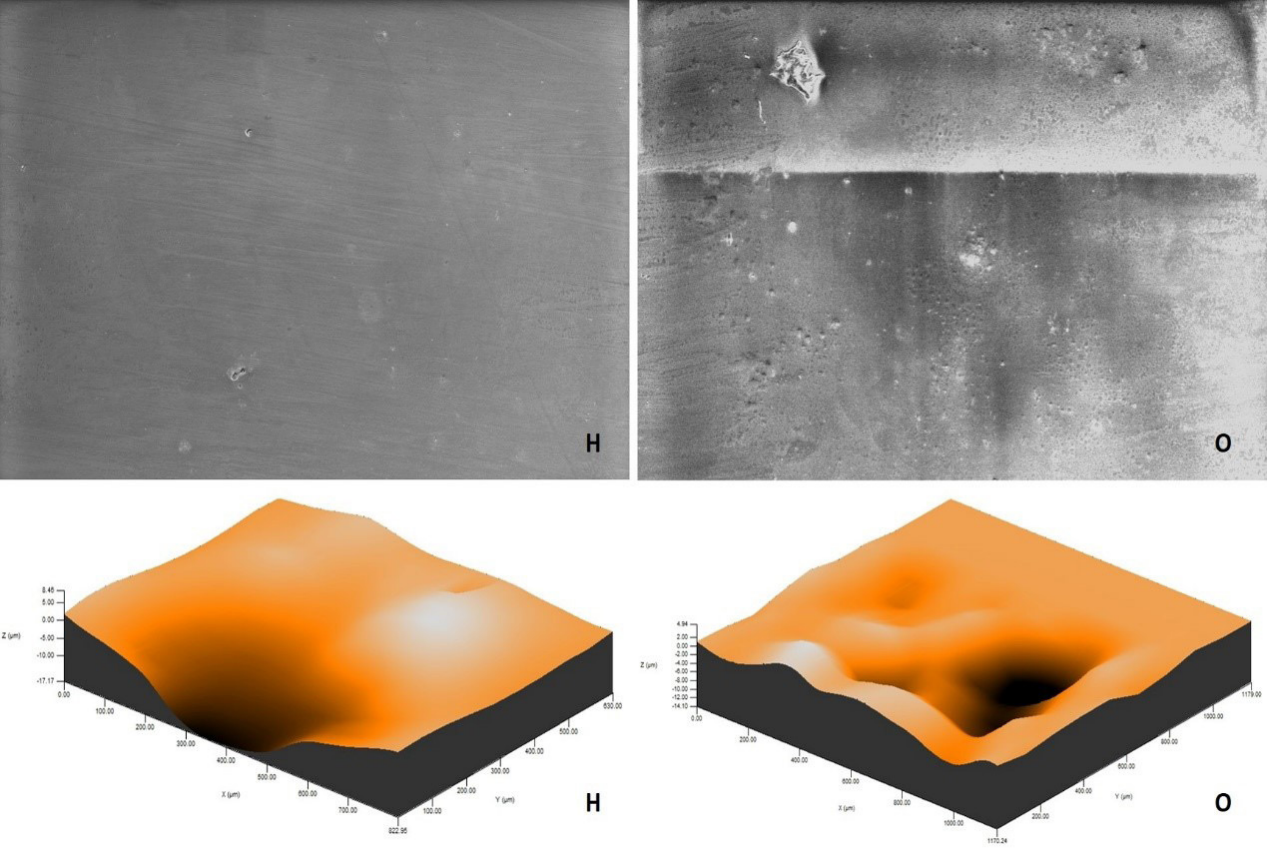
Environmental scanning electron microscope and 3D images of surface topography in group G specimens from the IP group. Original magnification × 1000 (H: At-home bleaching, O: In-office bleaching)

**Figure 2:**
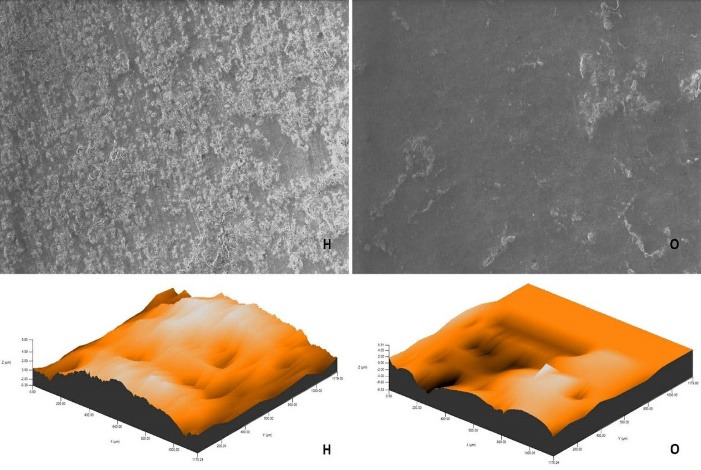
Environmental scanning electron microscope and 3D images of surface topography in group M specimens from the IP group. Original magnification × 1000 (H: At-home bleaching, O: In-office bleaching)

**Figure 3:**
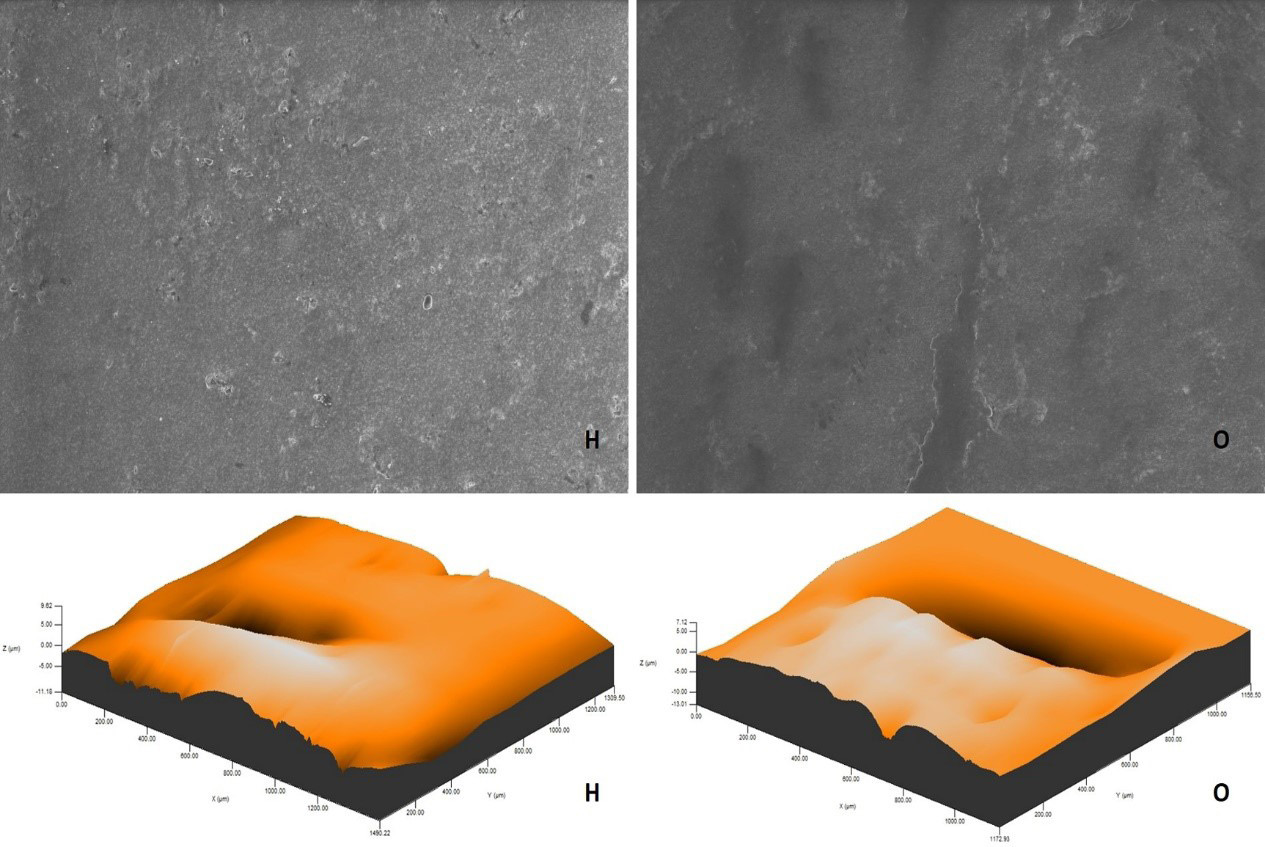
Environmental scanning electron microscope and 3D images of surface topography in group O specimens from the IP group. Original magnification × 1000 (H: At-home bleaching, O: In-office bleaching)

**Figure 4:**
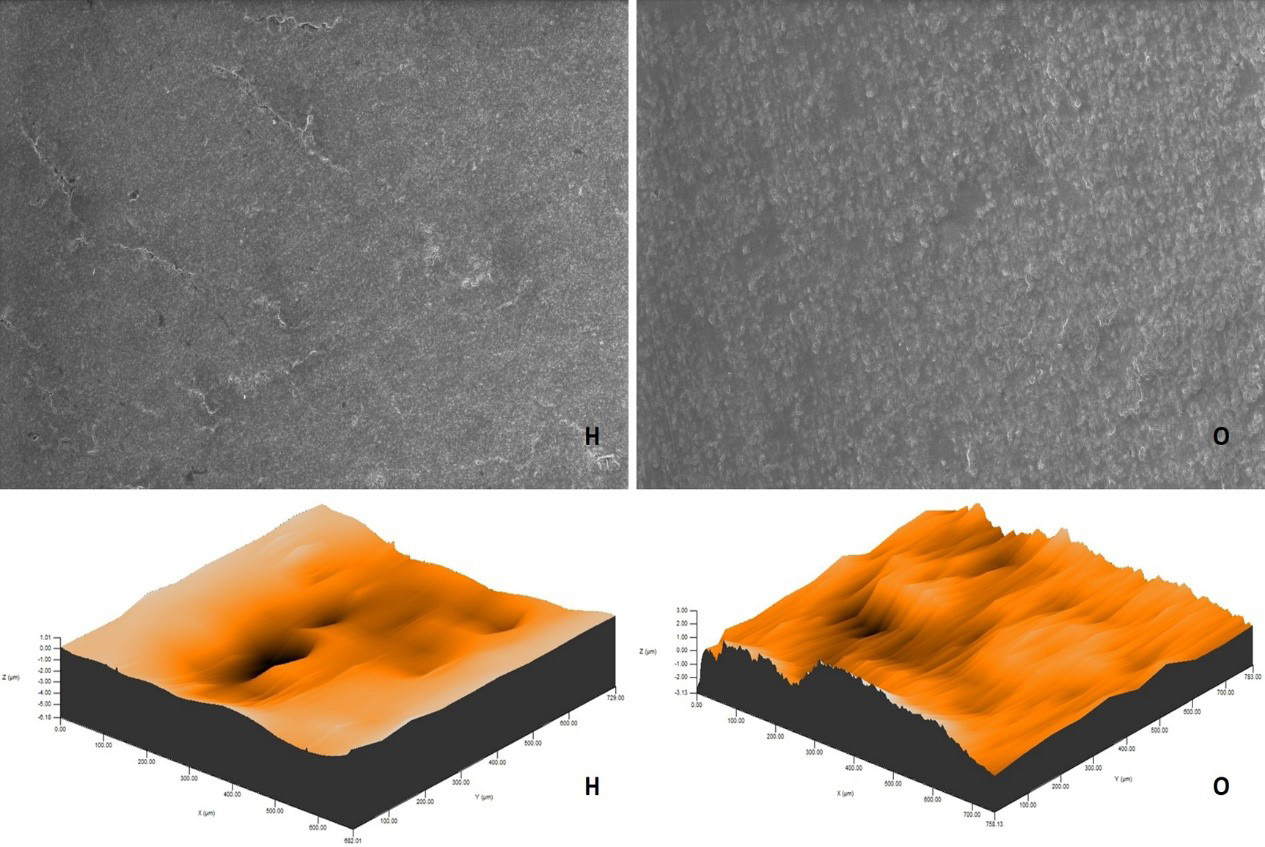
Environmental scanning electron microscope and 3D images of surface topography in group D specimens from the IP group. Original magnification × 1000 (H: At-home bleaching, O: In-office bleaching)

**Figure 5:**
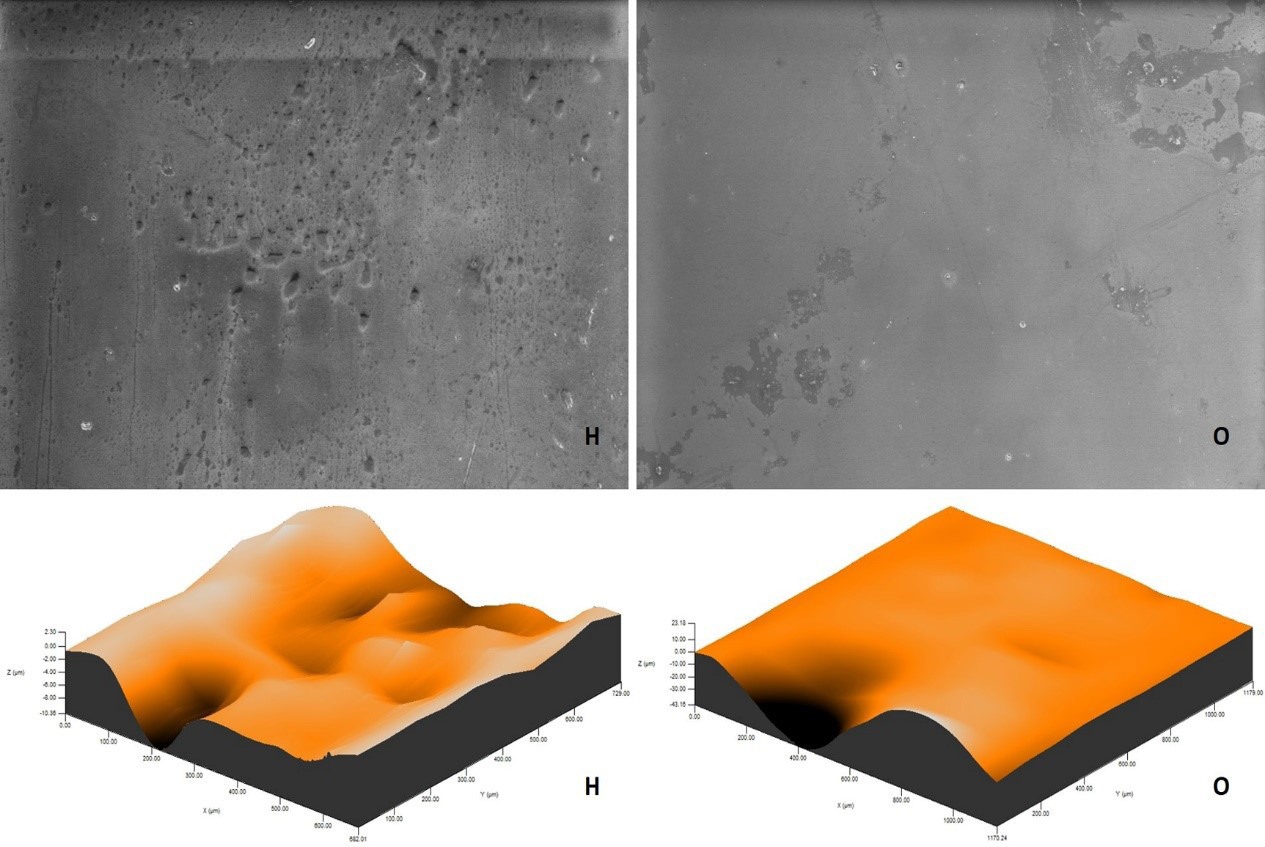
Environmental scanning electron microscope and 3D images of surface topography in group G specimens from the IC group. Original magnification × 1000 (H: At-home bleaching, O: In-office bleaching)

**Figure 6:**
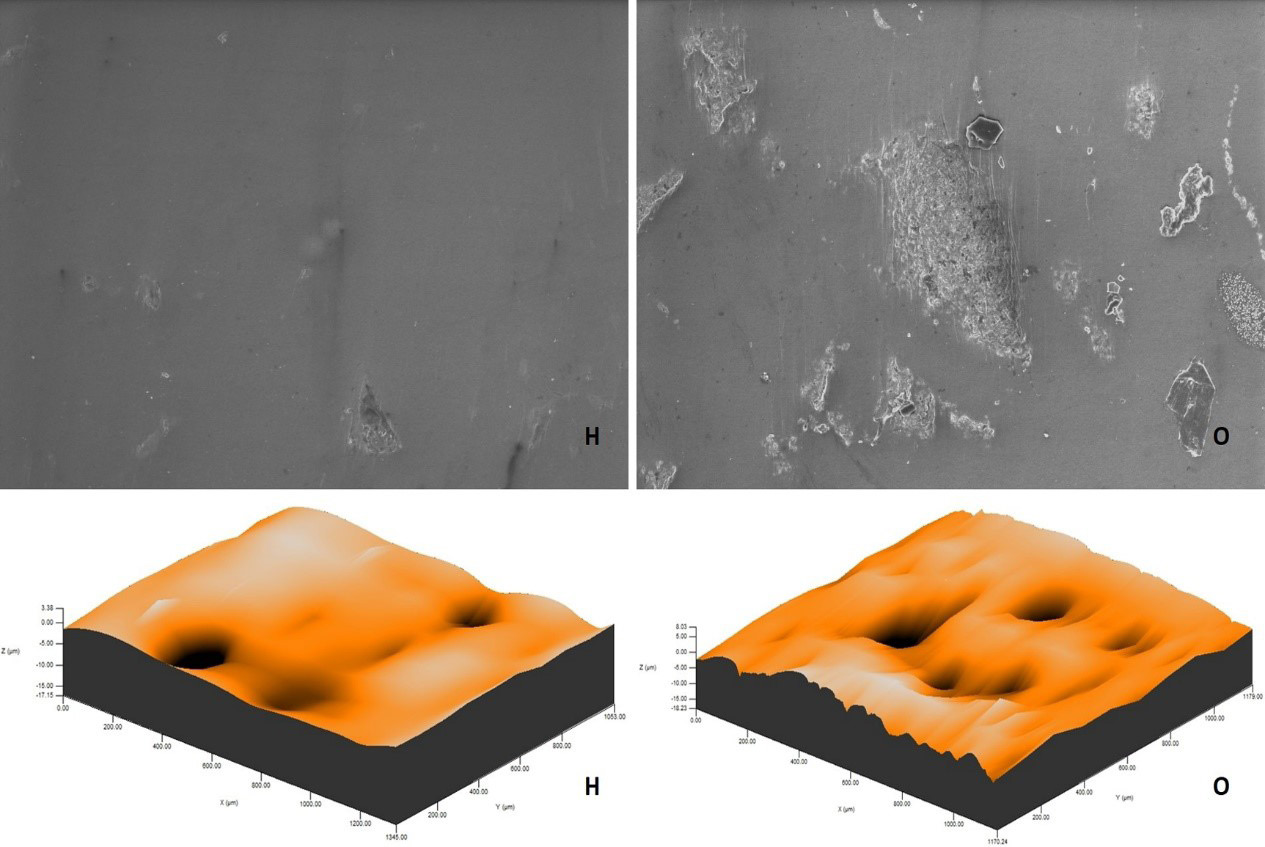
Environmental scanning electron microscope and 3D images of surface topography in group M specimens from the IC group. Original magnification × 1000 (H: At-home bleaching, O: In-office bleaching).

**Figure 7:**
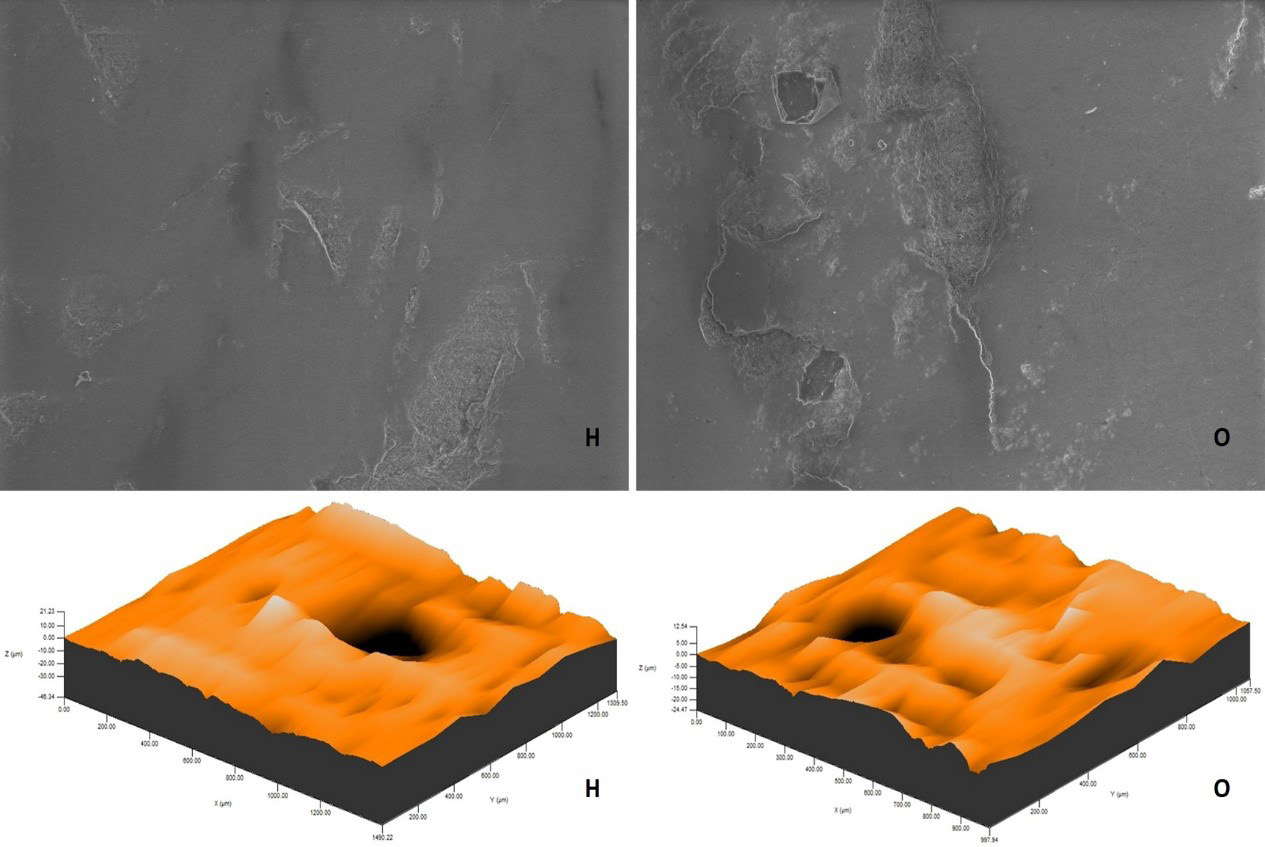
Environmental scanning electron microscope and 3D images of surface topography in group O specimens from the IC group. Original magnification × 1000 (H: At-home bleaching, O: In-office bleaching)

**Figure 8:**
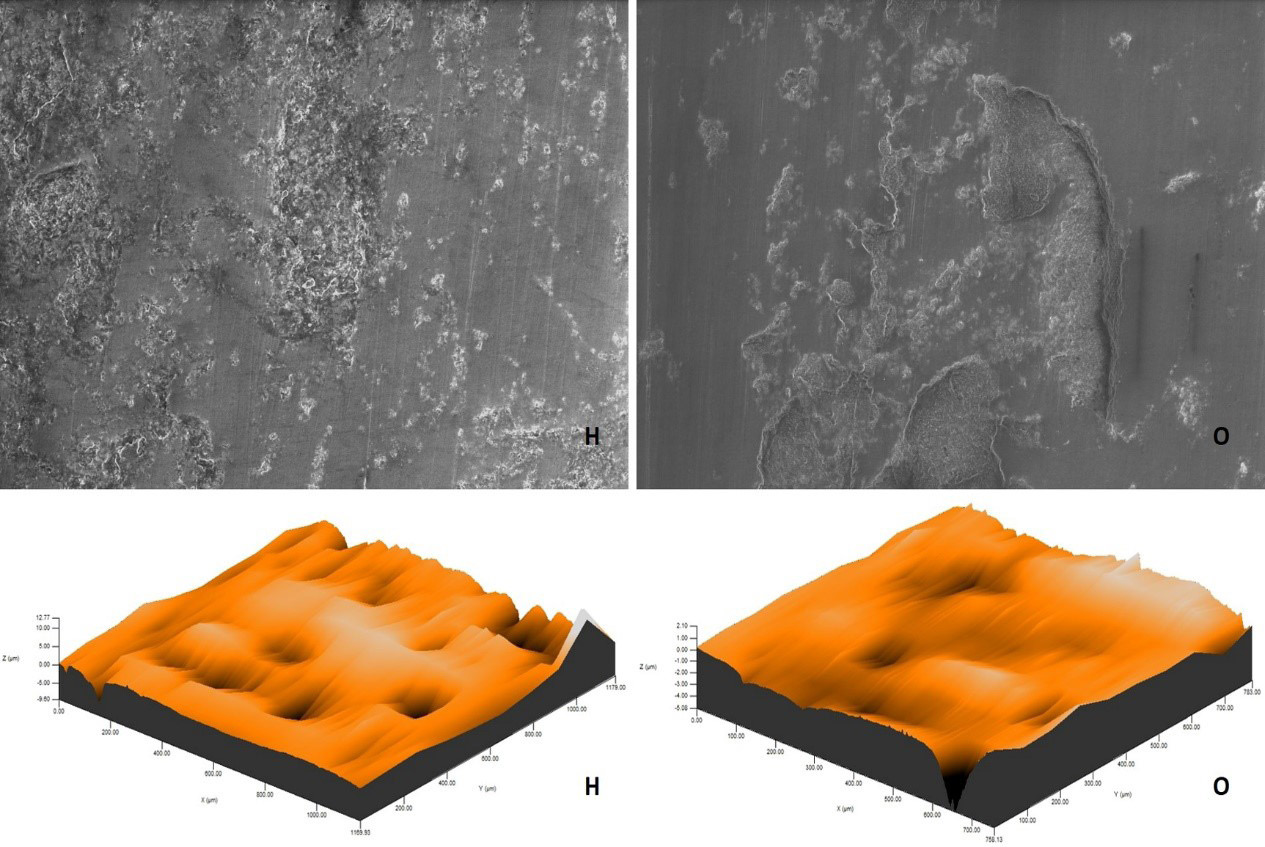
Environmental scanning electron microscope and 3D images of surface topography in group D specimens from the IC group. Original magnification × 1000 (H: At-home bleaching, O: In-office bleaching)

The roughness measurements and 3D surface topographies of the specimens whose color measurements were completed were made with a 0.008 mm cutoff in a contact scanning profilometer (Tencor P-7 Stylus Profiler; KLA-Tencor, Milpitas, CA, USA) that was capable of up to 360° measurements. Measurements were repeated three times for each specimen, and the average of these three measurements was recorded. Figures 1 to 8 show the 3D topography and ESEM images of the specimens at × 1000 magnification.

###  Statistical analysis

 Statistical analyses of the data were performed with a statistical software program (IBM SPSS Statistics, v20; IBM Corp, Armonk, NY, USA). Analyses included the Kolmogorov-Smirnov test for a normality test and an analysis of variance (ANOVA). The Tukey multiple comparison tests were used to compare the group means (α = 0.05).

## Results

Table 3 shows the results of the ANOVA, and Table 4 shows the mean and standard deviation values.

**Table 3:** ANOVA for color and surface roughness

	**Color**				**Surface roughness**		
	***df***	**Mean square**	***F***	***P***	**Mean square**	***F***	***P***
Material	1	5.367	1.525	0.219	4.612	3.878	0.051
Polishing	3	1.967	0.559	0.643	17.355	14.595	< .001
Bleaching	1	0.121	0.034	0.853	2.622	2.205	0.140
Material*Polishing	3	6.344	1.803	0.150	2.728	2.294	0.081
Material*Bleaching	1	0.595	0.169	0.681	2.288	1.924	0.168
Polishing*Bleaching	3	1.771	.503	0.681	1.280	1.076	0.362
Material*Polishing*Bleaching	3	4.091	1.163	0.327	0.642	0.540	0.656
Error	128	3.519			1.189		

**Table 4:** Least square means and standard deviation (SD) for color and surface roughness

**Material**	**Polishing**	**Bleaching**	**Color (∆E)**	**Surface roughness (µm)**	**N**
			**Mean±SD**	**Mean±SD**	
IPS e.max Press	Glaze	Home	1.12 ± 0.57	2.39 ± 0.95	9
		Office	1.11 ± 0.47	3.01 ± 1.45	9
	Meisinger	Home	1.76 ± 1.71	2.51 ± 1.02	9
		Office	0.99 ± 0.34	2.96 ± 1.68	9
	OptraFine	Home	1.65 ± 0.96	2.18 ± 0.82	9
		Office	1.54 ± 0.94	1.60 ± 1.15	9
	D + Z	Home	2.29 ± 1.67	1.61 ± 0.83	9
		Office	2.88 ± 2.54	1.06 ± 0.75	9
IPS e.max CAD	Glaze	Home	1.64 ± 1.36	2.82 ± 1.15	9
		Office	2.67 ± 1.04	2.51 ± 1.38	9
	Meisinger	Home	1.79 ± 1.16	3.33 ± 1.11	9
		Office	2.79 ± 1.47	2.77 ± 1.09	9
	OptraFine	Home	2.48 ± 1.84	3.37 ± 1.25	9
		Office	1.49 ± 0.95	2.71 ± 0.51	9
	D + Z	Home	1.93 ± 1.28	1.61 ± 0.83	9
		Office	1.64 ± 0.42	1.06 ± 0.75	9

The ANOVA showed no significant differences in the interaction of material*polishing*bleaching, polishing*bleaching, material*bleaching, and material*polishing on color and surface roughness (*P* > 0.05). According to the Tukey multiple comparison tests, there were no differences between the materials, polishing kits, and bleaching procedures (*P* > 0.05) in the color measurement. There were no significant differences between the materials and bleaching procedures (*P* > 0.05); however, there were significant differences between the polishing kits (*P* < 0.001) concerning surface roughness. The D + Z polishing kit resulted in lower Ra values (1.33 ± 0.80 μm) than the other polishing kits.

ESEM examination of the IP specimens showed that the specimens undergoing at-home and at-office bleaching in group G had similar images. Although the specimens undergoing at-office bleaching in group M exhibited a smooth surface, there were areas of dense mesh structure spread over the entire surface in the specimens undergoing at-home bleaching. Unlike group M, the specimens undergoing at-office bleaching in group O showed a smoother surface. Although the specimens undergoing at-home bleaching had slightly indented areas, the specimens undergoing at-home and at-office bleaching in group D showed very similar images, with recessed areas spread over the entire surface (Figures 1-4).

ESEM examination of the IC specimens showed that in group G, the home-bleached specimen had slightly indented areas, while the specimen undergoing at-office bleaching had very smooth surfaces. In group M, the specimens undergoing at-home bleaching showed smooth surfaces, while the office-bleached specimens had scattered crater areas. In group O, the specimens undergoing at-home and at-office bleaching procedures exhibited smooth surfaces quite similar to each other. Finally, in group D, the specimens undergoing at-home bleaching contained many irregularly dispersed areas, while the specimen surfaces undergoing at-office bleaching had irregularly distributed large, crater-like areas (Figures 5-8).

## Discussion

 The current study investigated the effects of at-home and in-office bleaching agents on the surface roughness and color changes of IP and IC specimens treated with four different polishing procedures. According to the results, since the bleaching and polishing processes did not change the color and surface roughness of the specimens, the null hypothesis was accepted.

Roughness and color are important factors affecting the long-term success of restorative materials.^19,20^ During clinical applications, the polish of the restoration might be removed, resulting in changes in surface smoothness. In such cases, glazing or polishing is required for the aesthetics and long-term stability of the restoration.^21^ However, a smooth surface structure is very important for the color of the restoration because it reflects a greater amount of light than a rough surface.^22^ Various discs, polishing kits, and pastes are offered by manufacturers for mechanical polishing.^19^ It has been reported that there is no difference in the effect of laboratory and chair-side polishing methods on color stability.^23^ In one study, the effect of different polishing kits and rubbers on the surface roughness of different porcelain types was similar to the glazed specimens.^24^ In contrast, in another study, polishing methods were more unsuccessful in reducing surface roughness compared with the glazing process.^25^ Although there was no material*polishing*bleaching interaction in the current study, and there was no difference between the effects of polishing processes on color stability, there were differences in the effect on surface roughness. This difference was observed in group D, which exhibited the lowest surface roughness values. Perceptible and acceptable color changes, according to the CIEDE2000 system, are ΔE_00_ > 0.8 and ΔE_00_ ≤ 1.8.^18,26^ In the current study, the mean color change was found to be 1.67 ± 1.18 for the IP specimens and 2.05 ± 1.47 for the IC specimens. Although color changes were acceptable for IP specimens, IC specimens were beyond this limit. The effect of at-home (1.83 ± 1.55) and in-office (1.89 ± 1.75) bleaching on color change was beyond the acceptable limits. In surface polishing processes, the mean values of the G (1.63 ± 1.1) and O (1.79 ± 1.62) groups were below the acceptable limits, while the mean values of the M (1.83 ± 1.38) and D (2.19 ± 1.88) groups were beyond them.

Bleaching agents may cause deterioration within the material by changing the structural and mechanical properties of the restorative material.^27^ Some studies^20,28-30^ have examined the effect of bleaching processes on the surface properties of ceramic and LDGC materials. In the literature, surface roughness values are between 0.55 and 1.68 µm for IP^31-33^ and 0.2 and 4.75 µm for IC.^34-38^ In the current study, mean surface roughness values were 2.16 ± 1.15 µm for IP specimens and 2.52 ± 1.25 µm for IC specimens. Demir et al^34^ reported that home bleaching applied with 16% carbamide peroxide for seven days increased the surface roughness of IPS e. max CAD specimens. While the Ra value of the control group was (0.59 ± 0.21 µm), the Ra value of the bleaching group was (1.09 ± 0.24 µm). Tinastepe et al^39^ reported that bleaching with 15% Opalescence PF (Ultradent) and 6% Opalescence Go (Ultradent) did not affect the color changes and roughness of IC specimens. In the current study, there were no significant differences in roughness and color between the bleaching processes in all double and triple interactions and between themselves, consistent with the study by Tinastepe et al.^39^

 In at-home bleaching, the bleaching agent may even contact the restorative material as it is not under the direct supervision of the professional. However, during in-office bleaching, as the operator applies the bleaching agent by considering only the patient’s natural teeth, the operator can prevent contact between the bleaching agent and ceramics. Therefore, until the effects of bleaching agents on ceramics are fully understood, in-office bleaching may be safer for patients with restorations in their oral cavities.

 One of the limitations of this study is that it was performed in vitro. Another limitation is that in addition to the surface roughness measurements of the specimens, bacterial adhesion was not included.

## Conclusion

 Based on the findings of this in vitro study, the following conclusions were drawn:

1. Bleaching and polishing processes did not adversely affect the roughness and color changes of LDGCs produced by press and CAD-CAM techniques.
2. Although there were no statistically significant differences between materials, polishing processes, and bleaching methods in terms of color changes, based on the tests performed, many Delta E values exceeded the 50:50% perceptibility threshold, which would certainly have clinical significance.
3. Although there was no significant difference between the materials and bleaching methods in terms of surface roughness, the D + Z polishing kit produced the lowest roughness values regardless of the material and the bleaching technique.

## Funding

 The study was supported by the Department of Scientific Research Projects (THD-2021-9220),

## Ethics Approval

 The ethics committee of faculty of dentistry, Ataturk university, Erzurum, Turkey approved this study (11.02.2021-02/12).

## Competing Interests

 The authors declare no conflict(s) of interest related to the publication of this work.
